# Anti-PD-1 immune checkpoint inhibitor-induced cardiotoxicity is associated with dysfunctional metabolism, muscle wasting and autophagy

**DOI:** 10.1038/s41598-025-34379-4

**Published:** 2026-01-15

**Authors:** Louisa Tichy, Traci L. Parry

**Affiliations:** Department of Kinesiology, University of North Carolina, Greensboro 1408 Walker Ave, Greensboro, NC 27412 USA

**Keywords:** Immunotherapy, Cardiotoxicity, Immune checkpoint inhibitor, Cancer, Anti-PD-1, Cancer, Cardiology, Cell biology

## Abstract

Immune checkpoint inhibitors (ICIs)have significantly improved overall survival rates in many aggressive cancers. Despite the recent clinical success, a rapidly increasing number of patients suffer from ICI-induced cardiotoxicity with often fatal outcomes, nonspecific symptoms and uninvestigated underlying pathological mechanisms. Therefore, this study explored metabolic, muscle wasting, and autophagic pathways and their roles in ICI-induced cardiac remodeling and dysfunction. Female C57BL/6 wildtype and LC3 transgenic (autophagy reporter) mice were randomly assigned to control (CON) and ICI-treated (ICI) groups. Mice underwent 4 weeks of ICI treatment (200 µg/mouse, intraperitoneally, twice/week). Echocardiography assessed ICI-induced changes in cardiac structure and function. At euthanasia, cardiac tissue was collected for Western Blot analysis of metabolic and muscle wasting signaling pathways and confocal fluorescent microscopy of autophagic flux. ICI treatment reduced tumor burden (-48% mass, *P* < 0.05) and led to significantly decreased cardiac function (-20%) and remodeling, including left ventricular dilation (+ 50%) and thinning of posterior cardiac walls (*P* < 0.05), indicative of dilated cardiomyopathy. Exploratory protein level analysis revealed dysfunctional muscle wasting (Atrogin1, MuRF1) disrupted AKT and FoxO1 signaling, and altered autophagic flux (*P* < 0.05). In this model, ICI-induced cardiotoxicity was characterized by severe cardiac remodeling and dysfunction, associated with dysfunctional metabolism, muscle wasting, and autophagy. To our knowledge, this is one of the first studies to explore underlying pathological mechanisms, adding novel and impactful insight to the still unclear characteristics of ICI-induced cardiotoxicity and supporting the critical need to further investigate side effects of immunotherapies to optimize clinical treatment of cancers.

**Supplementary Information:**

The online version contains supplementary material available at 10.1038/s41598-025-34379-4.

## Introduction

Lung cancer, specifically non-small cell lung cancer (NSCLC), remains the leading cause of cancer-related deaths worldwide^1,2^. The overall survival rate for patients with NSCLC treated with chemotherapy as the first line treatment is as low as 5%, which is well below most other cancers^3,4^. However, since first approval by the FDA in 2011, immune checkpoint inhibitors (ICIs) have significantly improved long-term remission and overall survival rates in many aggressive cancers that have previously been difficult to treat, such as NSCLC (up to 30% 5-year overall survival rate)^5,6^. ICIs can aid in overcoming tumor-manipulated immunosuppression and initiate a biological immune response to promote tumor cell destruction^7^. In addition to improving overall survival and long-term remission rates, ICIs are generally associated with a preferable and less severe side effect profile compared to other anti-cancer therapies, such as chemotherapy or tyrosine kinase inhibitors^3^.

However, despite the positive clinical outcomes of this new anti-cancer immunotherapy, an increasing number of cancer patients treated with ICIs develop and suffer from ICI-induced cardiotoxicity with the highest mortality rates of all ICI-associated immune related adverse events (irAEs; 36–67% mortality)^8–10^. While clinical cases of ICI-induced cardiotoxicity are rising rapidly, awareness of this severe side effect is low and clinical manifestations and underlying molecular mechanisms of this phenomenon are largely unknown. Myocarditis and new arrhythmias diagnosed *via* electrocardiography are the most recognized forms of ICI-induced cardiotoxicity, and are speculated to involve cardiac remodeling and dysfunction^8,9,11^. Initially reported incidence rates ranging from 0.06 to 1% are likely underestimated due to varying clinical manifestations and overall lack of awareness of this condition.

Electrocardiography is often used to identify underlying dysfunction/arrhythmias, such as atrioventricular block, atrial and/or ventricular premature contractions, and atrial fibrillation^8,12^. Unfortunately, analyses of underlying structural and functional changes *via* echocardiography are limited^9,13^. Increased pro-inflammatory cytokine production and upregulated inflammatory pathways are likely involved in the molecular mechanisms of ICI-induced cardiotoxicity due to the nature of this type of immunotherapy to initiate a biological immune response^14^. Further investigation is crucial to elucidate if other cellular mechanism are involved in the underlying pathology of ICI-induced cardiotoxicity, such as abnormal metabolism and muscle wasting resulting in cardiac dysfunction. Dysfunctional metabolic pathways, such as the AKT/FoxO pathway, and abnormally increased muscle wasting, through the ubiquitin-proteasome and autophagy-lysosome degradation systems (i.e., expression of MuRF1 and Atrogin1), have been strongly associated with anthracycline chemotherapy-induced cardiotoxicity, the most extensively studied cardiac pathology associated with anti-cancer therapies^15^. Thus, these pathways may also be involved in ICI-induced cardiotoxicity and could aid in identifying potential therapeutic targets to overcome this severe side effect. As such, there is a critical clinical need to identify and characterize ICI-associated cardiotoxicity to optimize the use of ICI treatment of human cancers. Therefore, the purpose of this study was to explore metabolic (AKT/FoxO), muscle wasting and autophagic (MuRF1, Atrogin1) pathways and their roles in ICI-induced cardiac remodeling and dysfunction in female mice. This study focuses on female mice due to recent clinical and clinical observations have identified increased risk of development of ICI-induced cardiotoxicity and increased mortality rates in females^10,16,17^.

## Methods

### Research design

Female LC3 Transgenic (C57BL/6-Tg(CAG-RFP/EGFP/Map1lc3b)1Hill/J; Jackson Laboratory strain #027139) and age/sibling matched C57BL/6 wildtype mice (~ 12–14 weeks old; adulthood) were used as the preclinical model in this study^10,16,17^. Groups (outlined below) contained equal amounts of LC3 transgenic (autophagy reporter mice for measurement of autophagic flux, at least 3 mice per group) and wildtype mice. Mice were housed in standard mouse cages and bred onsite at the University of North Carolina Greensboro. Rooms in the animal facility were temperature-controlled with a 12:12 light-dark cycle and equipped with *ad libitum* access to water and standard rodent chow diet. All procedures and experiments comply with the Animal Welfare Act guidelines and the ARRIVE guidelines and were approved by the University of North Carolina Greensboro’s Institutional Animal Care and Use Committee.

Mice were randomly selected and separated into non-ICI controls (CON; *n* = 5) and ICI-treated (ICI; *n* = 5) groups. Mice underwent a 4-week ICI treatment protocol (see ICI Treatment section). Mice were euthanized on day 28 by isoflurane overdose followed by cervical dislocation. Cardiac tissue was collected, weighed and flash frozen in liquid nitrogen or embedded in optimal cutting temperature gel (OCT) and frozen on dry ice.

### Tumor model

A tumor model was included in this study design as proof-of-concept for the clinical relevance and efficacy of the ICI protocol. Lewis Lung Carcinoma cells (LLC; ATCC: CRL-1642, Manassas, VA, USA) were grown in Dulbecco’s Modified Eagle Medium (DMEM: ATCC; 30 − 002, Manassas, VA, USA) supplemented with 10% Fetal Bovine Serum (FBS), in an incubatory set to 5% CO2 and 37° Celsius. The LLC model is well characterized in the literature and grows a subcutaneous tumor (under the skin) to allow for easily measurable tumor growth and evaluation of tumor progression^18,19^. T groups were inoculated subcutaneously in the right flank with a concentration of 5 × 10^5^ LLC cells in 100 µl of sterile phosphate buffered saline (PBS). Tumor length, width, and thickness was measured by using a Vernier caliper. Tumor measurements were used to calculate relative tumor mass and tumor volume using the following formulas, in which a equals the longest diameter and b equals the shortest diameter of the tumor: (1) relative tumor mass = wet tumor mass/(total body mass - wet tumor mass); (2) estimated tumor volume (mm^3^) = (a*b^2)/2. Tumor bearing mice were not included in any statistical analyses other than analyses of tumor characteristics.

### Immune checkpoint inhibitor treatment

More than 50% of patients who develop ICI-induced cardiotoxicities are treated with anti-PD-1 as the type of ICI^20–22^. Therefore, the present study featured PD-1 specific antibody (BioXCell) as the ICI immunotherapy protocol. The effectiveness and potential side effect profile of this ICI treatment is comparable to human ICI treatment^13,23^. Anti-PD-1 was diluted in PBS to a dose of 200 µg per mouse^13,24,25^. Throughout the 4 week protocol, mice in the ICI groups were injected intraperitoneally with Anti-PD-1 treatment (200ug/mouse) twice each week^24,25^. For the ICI + T group, ICI injection day 1 occurred three days post tumor inoculation. To control for injection-associated stressors, non-ICI groups received saline placebo injections. Herein after, the specific anti-PD-1 treatment of this study will be referred to as “ICI”.

### Cardiac structure and function

To assess ICI-induced cardiac structural and functional changes, conscious echocardiography (GE Vivid 7 Dimension) was performed at baseline and euthanasia timepoints. In supine position, mice were restrained, and hair was removed from the chest *via* depilatory agent. Transduction jelly was applied to the chest and M-mode images were recorded in the parasternal long-axis view at the level of the papillary muscle. UltraLinq software (Durham, NC, USA) was used to analyze the echocardiograms and statistical analysis represent averages of three cardiac cycles of each mouse.

### Western blot

Approximately 35 mg of cardiac tissue per mouse was homogenized via 8 M Urea lysis buffer with a Qiagen TissueLyser LT homogenizer (Hilden, Germany). Homogenates were centrifuged at 12000G for 10 min at 4° Celsius and the supernatant was then collected for total protein analysis via Bradford method^26^. Based on Bradford results, 50ug/uL of protein per sample were used to make Western Blot samples. Samples were loaded into 4–12% BisTris graded gels (ThermoFisher; Waltham, MA, USA) in addition to MagicMark XP Western Protein Standard (ThermoFisher; Waltham, MA, USA) as molecular weight comparison. NuPAGE MES or MOPS running buffer (ThermoFisher; Waltham, MA, USA) were used to perform gel electrophoresis, proteins were transferred onto a polyvinylidene fluoride membrane and blocked in 5% non-fat milk. Protein contents were determined by incubation with antibodies against muscle wasting and atrophy pathways (anti-Atrogin1, 1:500 dilution in 5% milk [SC-166806]; anti-MuRF1, 1:500 dilution in 5% milk [SC-398608]), autophagic pathways (anti-p62, 1:500 dilution in 5% milk [CST8025]), and metabolic pathways (anti-AKT, 1:1000 dilution in 5% milk [CST9271]; anti-P-AKT, 1:1000 dilution in 5% milk [CST4060]; anti-FoxO1, 1:500 dilution in 5% milk [CST2880]; anti-FoxO3a, 1:500 dilution in 5% milk [CST12829]; anti-P-FoxO1/3a, 1:500 dilution in 5% milk [CST9464]). Primary antibodies were prepared in 10mL of 5% non-fat milk solution at 1:1000 to 1:250 concentration dilution. Membranes were incubated at 4° Celsius overnight, followed by incubation with species-specific secondary antibody. Membranes were developed in ECL and imaged via BioRad ChemiDoc (XRS+) imaging. Protein content was assessed by densitometry (Quantity One, v3.0) and normalized to a loading control, GAPDH (anti-GAPDH, 1:4000 dilution in 5% milk [G8795]).

### Autophagic flux via fluorescent microscopy

LC3 Transgenic mice (C57BL/6-Tg(CAG-RFP/EGFP/Map1lc3b)1Hill/J; Jackson Laboratory strain #027139) used in this study express dual tagged LC3 proteins with RFP (red fluorescent protein), GFP (green fluorescent protein). Fluorescent microscopy was used to analyze autophagic flux in LC3 transgenic mice by identifying different conformational stages of the dual LC3-GFP-RFP reporters in cardiac tissue. Early phase autophagosomes appear as yellow puncta, while late phase autophagolysosomes appear as red puncta via fluorescent microscopy. Cardiac tissue was embedded in OCT gel, cross-sectionally sliced by cryotome at 8–12 μm, and mounted on microscopy slides with Prolong Diamond Antifade (Invitrogen, cat.no. P36961) and cured for 24 h in the dark (per manufacturer instructions). Fluorescent microscopy was used to image 10 random fields of each heart at 40x (EVOS FL Inverted Microscope; ThermoFisher; Waltham, MA, USA). Excitation was set at 488 nm (GFP), 543 nm (RFP), and 405 nm (DAPI). All images were analyzed using the Green and Red Puncta Colocalization Macro for ImageJ^27^ to objectively analyze autophagosome and autophagolysosome content to measure autophagic flux.

### Equipment and settings

Conscious echocardiography was performed using a GE Vivid 7 Dimension instrument (GE Healthcare, Chicago, IL). All images were collected on conscious mice, lightly restrained, in a supine (Reverse Trendelenburg) position. M-mode images were recorded in the parasternal long-axis view at the level of the papillary muscle. Collected images were analyzed by UltraLinq software (Durham, NC, USA) on 3 consecutive cardiac cycles. For western blots, a BioRad ChemiDoc XRS + Imager (Hercules, CA). All groups were imaged at the same time under identical conditions (i.e., all blots for specific protein signal imaged at same time). Blots were developed by ECL in a range of 8 s to 360 s, based on clarity and brightness of bands. All images were visually assessed for overexposure using the “pseudo” function. Densitometry was performed using QuantityOne software (BioRad, Hercules, CA). Densitometry signals for each protein analyzed were normalized to corresponding GAPDH signals as a loading control from the same membrane. For fluorescent microscopy, images were collected at 40x from an EVOS FL Inverted Microscope (ThermoFisher; Waltham, MA, USA). During image collections, excitation was set at 488 nm (GFP), 543 nm (RFP), and 405 nm (DAPI). Images were collected from 10 random heart fields as single laser images (i.e., GFP only) as well as an overlay of all 3 lasers (GFP, RFP, DAPI). All images were objectively analyzed using the Green and Red Puncta Colocalization Macro for ImageJ (National Institute of Health)^27^. No touch up tools (i.e., Photoshop) were used. Processing (changes to image brightness/contrast) was kept to a minimum and only used to optimize the image for sake of data collection. If processing occurred, this was applied to the entire image so that all groups were equally affected.

### Statistical analyses

**Fig. 1:**
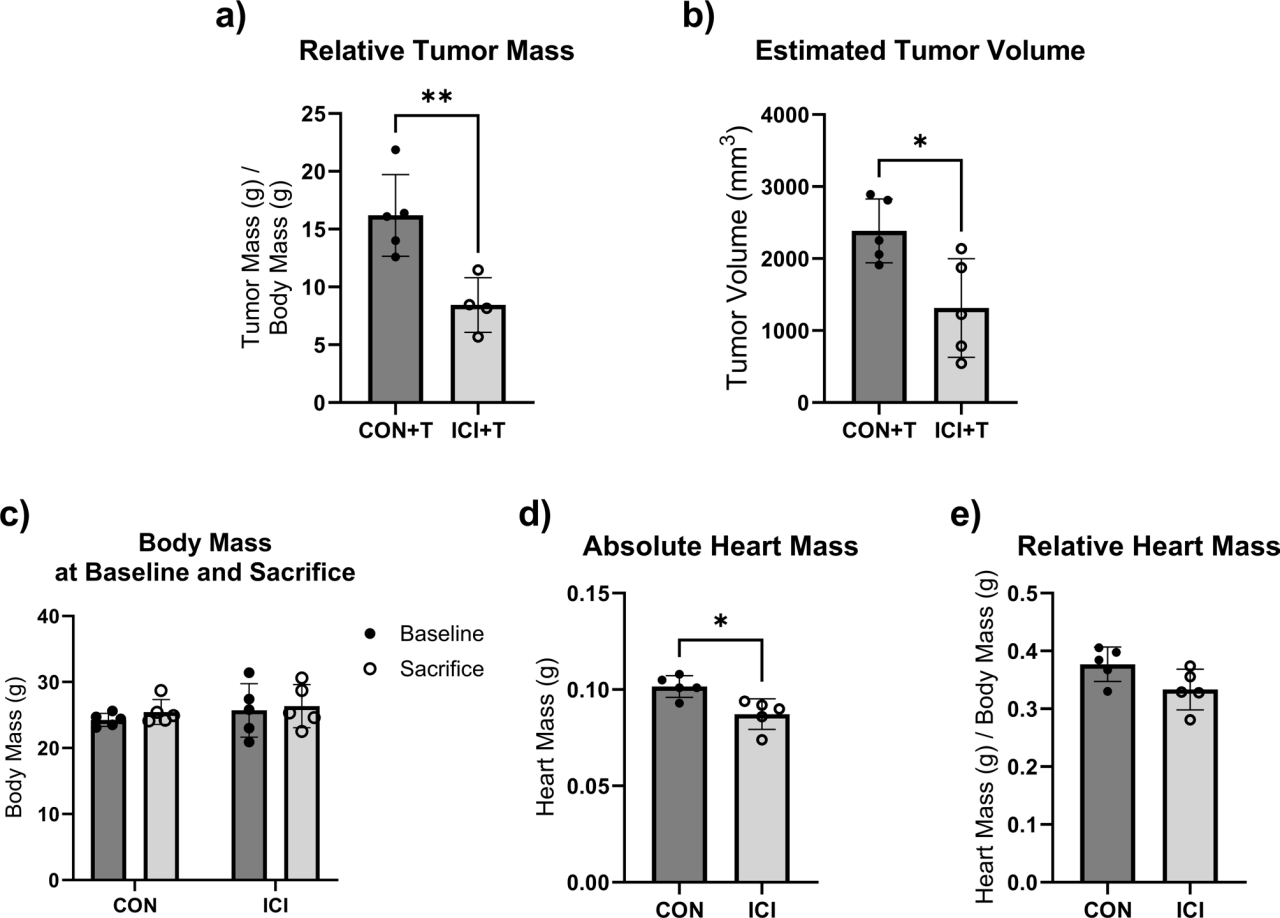
Tumor characteristics of control and ICI-treated tumor bearing mice (Fig. 1a, b), and body mass and heart mass of control and ICI-treated mice (Fig. 1c-e). Relative tumor mass (**a**) was calculated by normalizing wet tumor mass in grams to body mass in grams. Estimated tumor volume (**b**) was calculated by using the following formula: tumor volume (mm^3^) = (a * b)^2^/2, with a being the longest diameter and b being the shortest diameter of the tumor. Body mass (**c**) was weighed in grams at baseline and endpoint. Absolute heart mass (**d**) was weighed in grams and then normalized to body mass in grams (**e**). CON + T, control tumor bearing group (*n* = 5); ICI + T, ICI treated tumor bearing group (*n* = 4 in Fig. 1A excluding one animal due to difficulties during tumor excision, *n* = 5 in Fig. 1B); *N* = 9/10. CON, control group (*n* = 5); ICI, ICI treated group (*n* = 5); *N* = 10. Values are reported as mean ± SD. *Significant difference, *P* < 0.05; **Significant difference, *P* < 0.01.

**Fig. 2:**
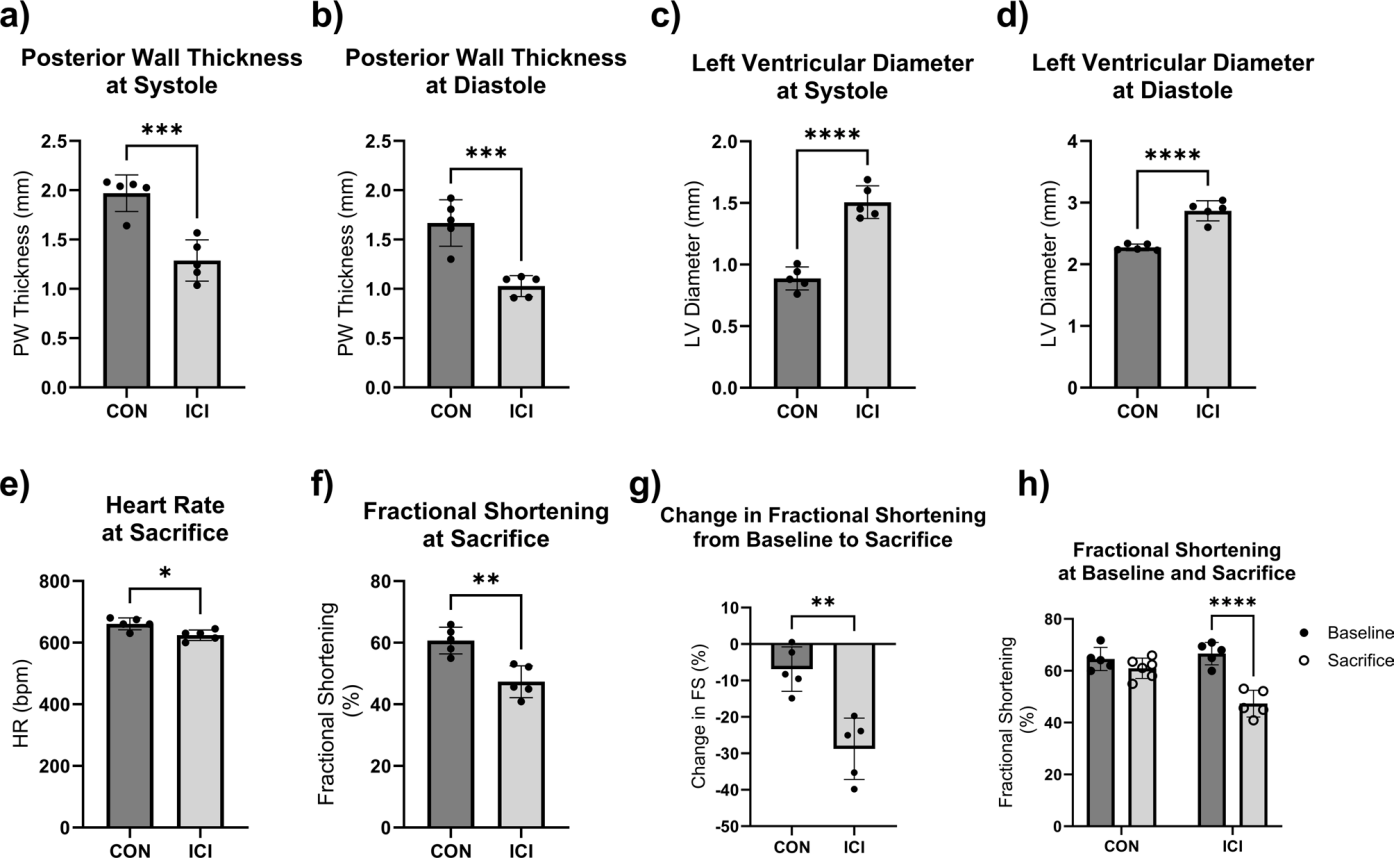
In Vivo Echocardiography at Study Endpoint. Wall thicknesses (**a**, **b**) and left ventricular diameter (**c**, **d**) at end-diastole and end-systole represent an average of three consecutive cardiac cycles per mouse. Fractional shortening as a measure of cardiac function (**e**-**g**) represents an average of three consecutive cardiac cycles per mouse. Change scores (**f**) were calculated by subtracting average baseline from average endpoint scores and dividing results by baseline scores. CON, control group (*n* = 5); ICI, ICI treated group (*n* = 5); *N* = 10. Values are reported as mean ± SD. *Significant difference, *P* < 0.05; ***Significant difference, *P* < 0.001; ****Significant difference, *P* < 0.0001.

**Fig. 3:**
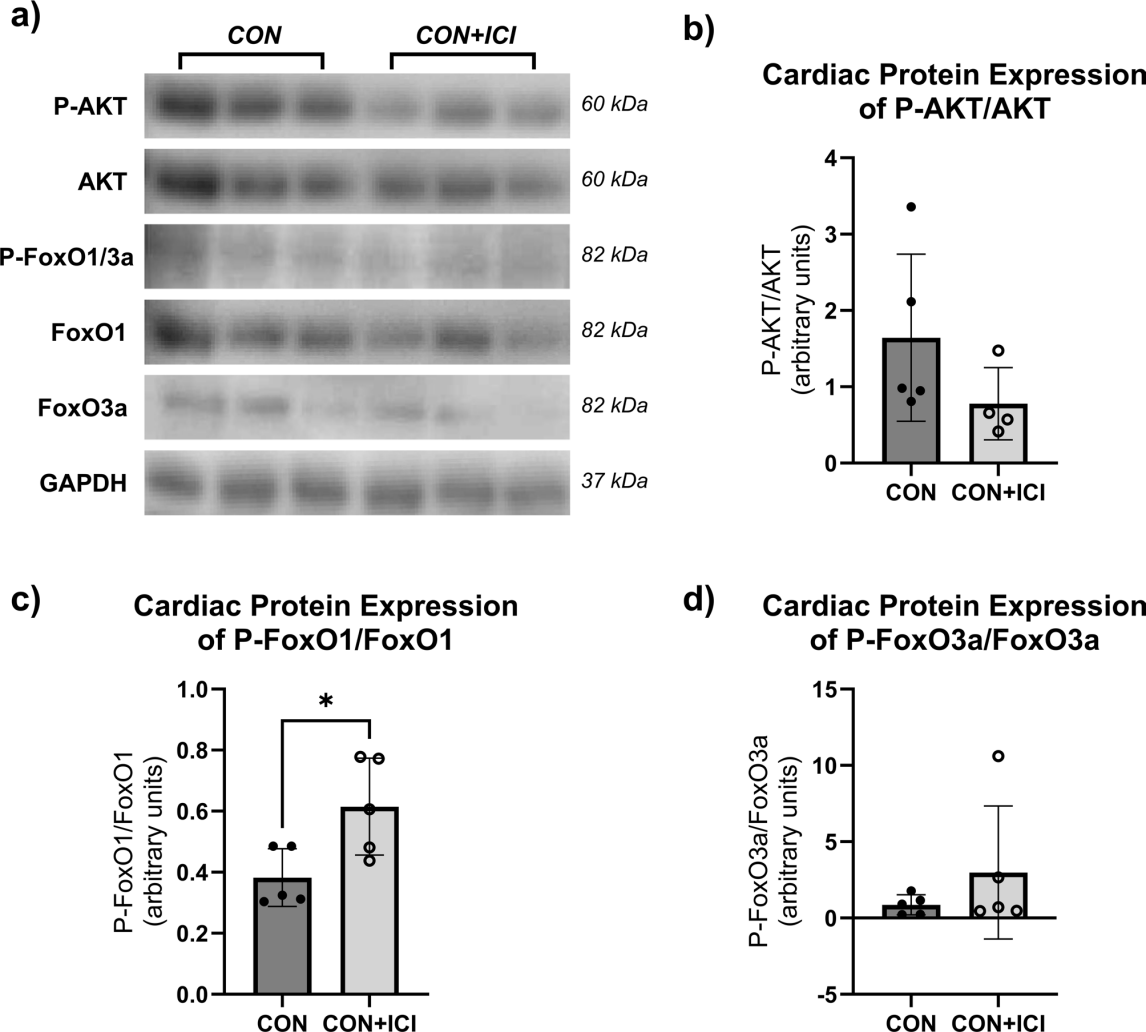
Effects of ICI treatment on protein levels of P-AKT (**b**), P-FoxO1 (**c**), and P-FoxO3a (**d**) in cardiac muscle. Cropped, representative western blot images (**a**); original blots are presented in supplementary files. CON, control group (*n* = 5); ICI, ICI treated group (*n* = 5). *N* = 10. Values are reported as mean ± SD. *Significant difference, *P* < 0.05.

**Fig. 4:**
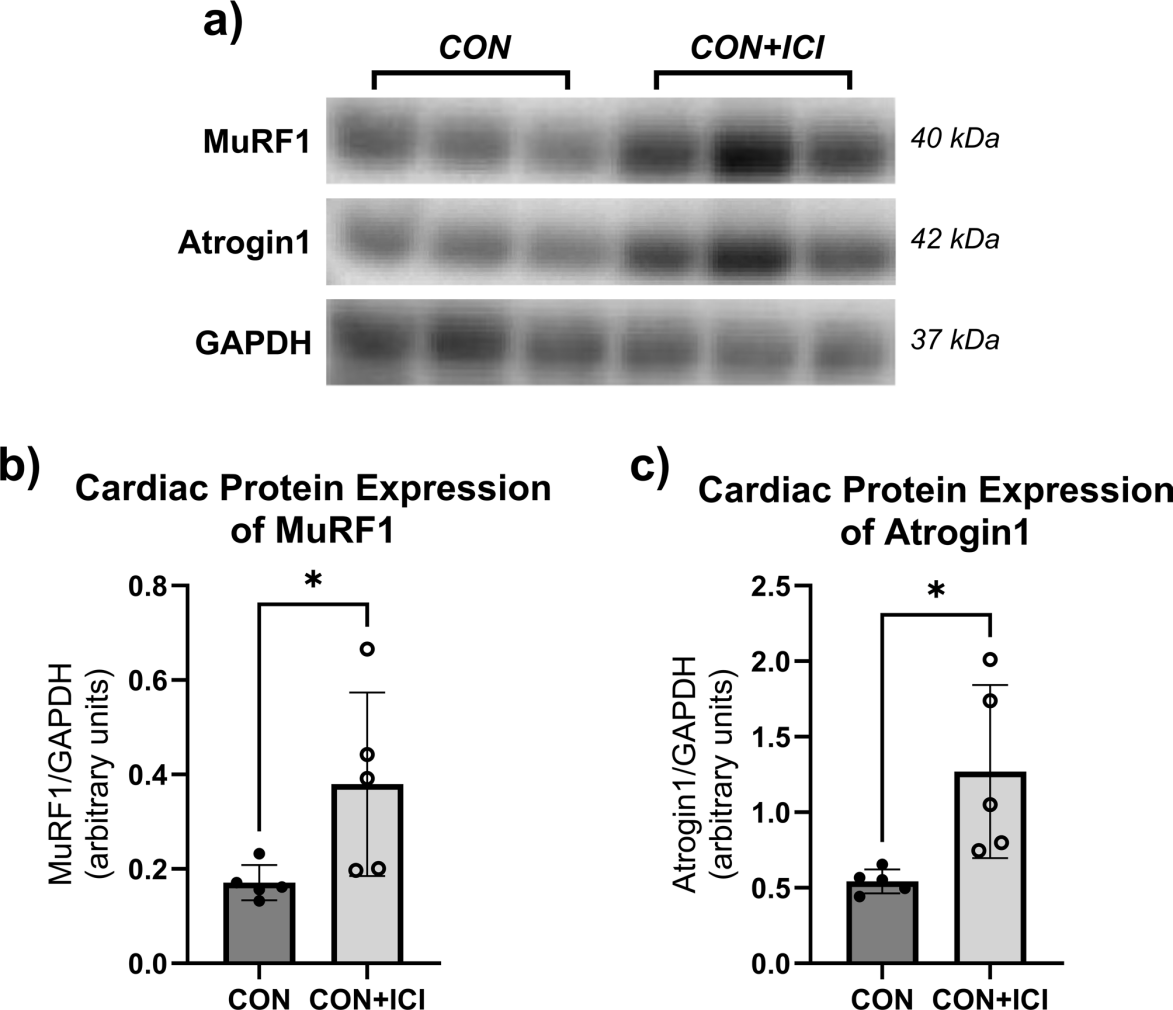
Effects of ICI treatment on protein levels of MuRF1 (**b**) and Atrogin1 (**c**) in cardiac muscle. Cropped, representative western blot images (**a**); original blots are presented in supplementary files. CON, control group (*n* = 5); ICI, ICI treated group (*n* = 5). *N* = 10. Values are reported as mean ± SD. *Significant difference, *P* < 0.05.

**Fig. 5:**
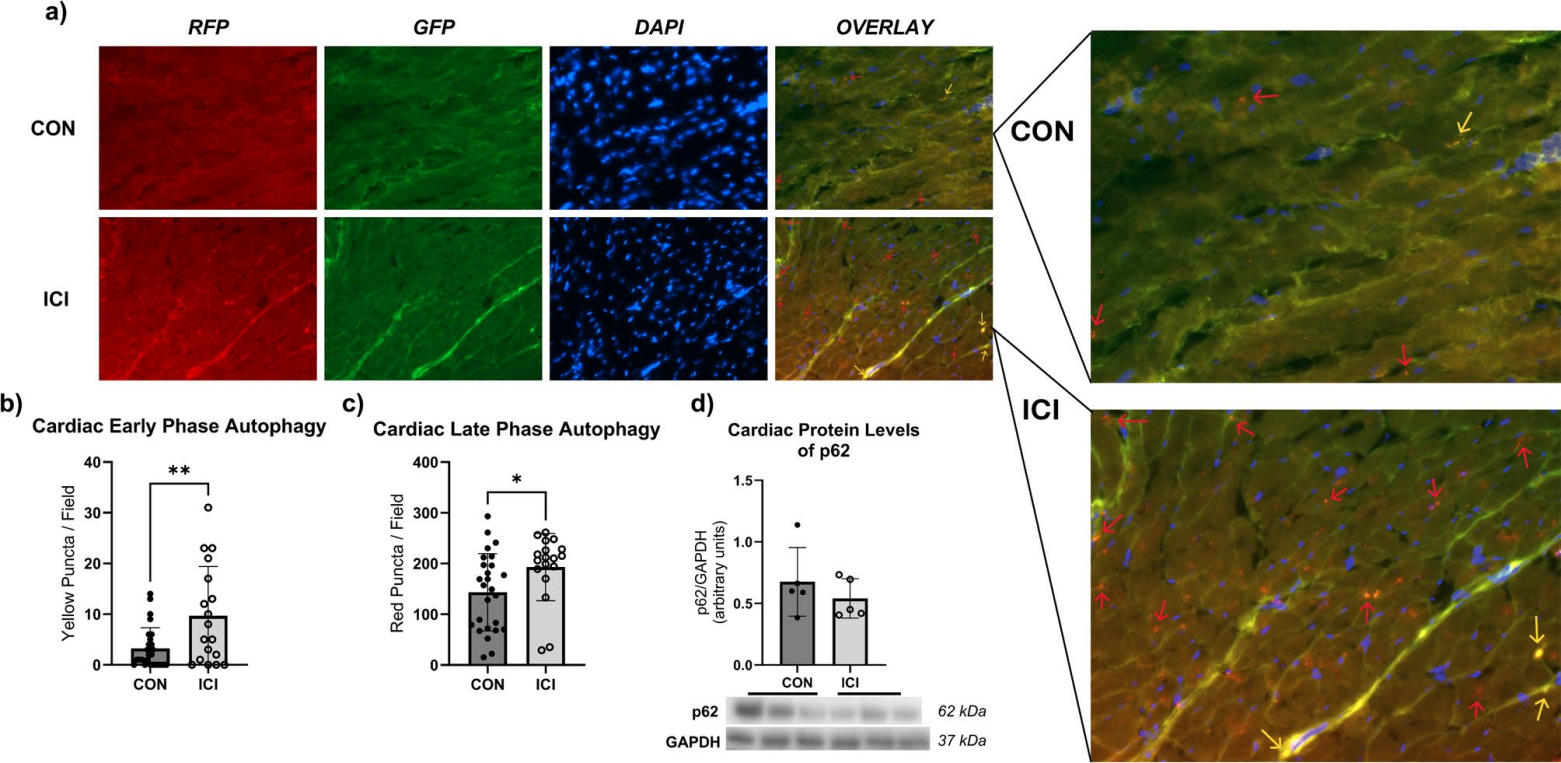
Autophagic flux analysis in cardiac muscle via Fluorescent Microscopy. Yellow puncta (**b**) represent early-phase autophagosomes. Red puncta (**c**) represent late-phase autolysosomes. Effects of ICI treatment on protein levels of p62 (**d**) in cardiac muscle. Representative fluorescent images have been taken at 40x magnification (**a**). Yellow arrows point towards yellow puncta/early-phase autophagosomes, red arrows point towards red puncta/late-phase autolysosomes (a). Cropped, representative western blot images (d); original blots are presented in supplementary files. CON, control group (*n* = 5); ICI, ICI treated group (*n* = 5); *N* = 10. Values are reported as mean ± SD. *Significant difference, *P* < 0.05. **Significant difference, *P* < 0.01.

Power analyses were performed to detect differences in cardiac structure and function between groups based on preliminary data at an alpha level of 0.05 set a priori according to current standards for significance. All data is presented as mean ± standard deviation (SD). Statistical software GraphPad Prism (v10.5; LaJolla, CA) was used for all statistical analyses. Shapiro-Wilk tests were performed due to small sample size and did not show evidence of non-normality for all analyzed variables. Further parametric statistical tests were performed following the results of the normality tests. Unpaired Student’s T-tests were performed to determine ICI efficacy via differences in relative tumor mass and estimated tumor volume between CON + T and ICI + T groups (Fig. 1a, b), determine the effects of ICI treatment on body mass (Fig. 1c), absolute and relative heart mass (Fig. 1d, e), to analyze the effect of ICI treatment on cardiac geometry measures (i.e., septal wall thickness (SW), posterior wall thickness (PW; Fig. 2a, b), left ventricular diameter (LVD; Fig. 2c, d) at systole (s) and diastole (d)) between CON and ICI groups, to analyze the effect of ICI treatment on fractional shortening as a measure of cardiac function (Fig. 2e-g) at systole and diastole between CON and ICI groups, and to compare differences in underlying cellular signaling pathways between CON and ICI groups (Figs. 3, 4 and 5). Repeated measures ANOVA was performed to analyze echocardiography measurements (i.e., cardiac structural and functional measures) at baseline and euthanasia within CON and ICI groups. Change scores were performed to calculate percent change in cardiac function from baseline to euthanasia. Tukey’s post hoc testing was performed to identify where significant differences occurred.

## Results

### ICI treatment efficacy

ICI treatment was administered to ICI groups twice each week throughout the 4-week study. While this study focused on the effects of ICI alone on cardiac structure, function and underlying signaling pathways, ICI treatment was also administered to tumor-bearing mice as a proof-of-concept of the clinical relevance and efficacy of the administered ICI protocol. Analyses revealed that ICI-treated tumor-bearing mice (ICI + T) showed significantly smaller tumors based on relative tumor mass (CON + T: 16.19 g ± 3.53 vs. ICI + T: 8.44 g ± 2.37; −48%; *p* < 0.05; Fig. 1a) and estimated tumor volume (CON + T: 2385mm^3^ ± 443.3 vs. ICI + T: 1314mm^3^ ± 684.2; −45%; *p* < 0.05; Fig. 1b) compared to CON + T mice. These data indicate that the administered ICI treatment protocol resulted in significant reductions in tumor burden and therefore supports the efficacy and clinical relevance of this protocol.

### ICI is associated with cardiac remodeling and dysfunction

While ICI treatment did not affect body mass (Fig. 1c) or relative heart mass (1e), absolute heart mass of ICI treated mice was significantly smaller compared to CON mice (*p <* 0.05; Fig. 1d). Baseline measurements (see supplemental material) indicated no significant difference detected between CON and ICI groups in SW, PW, or LVD measurements at systole or diastole.

While endpoint SW thicknesses at systole and diastole were not significantly different between the groups, PW and LVD cardiac geometry measures were significantly different at euthanasia (*p* < 0.05; Fig. 2). LVD measurements were significantly larger in the ICI group compared to CON counterparts at both systole (CON: 0.89 mm ± 0.08 vs. ICI: 1.5 mm ± 0.13; +69%) and diastole (CON: 2.28 mm ± 0.05 vs. ICI: 2.87 mm ± 0.16; +26%; *p* < 0.05; Fig. 2c, d), indicating dilation of the left ventricle at relaxation and contraction phases of the heart. PW thickness was significantly smaller in ICI mice compared to CON mice both at systole (CON: 1.98 mm ± 0.17 vs. ICI: 1.29 mm ± 0.21; −35%) and diastole (CON: 1.67 mm ± 0.21 vs. ICI: 1.03 mm ± 0.11; −38%; *p* < 0.05; Fig. 2a, b), indicating thinning of the posterior walls. These data suggest that ICI administration led to dilation of the left ventricle and thinning of the posterior walls, which suggests weakening of the cardiac muscle and a phenotype consistent with dilated cardiomyopathy.

In vivo echocardiography assessments were also used to identify changes in cardiac function due to ICI treatment. No significant difference in fractional shortening and heart rate at baseline was detected between CON and ICI groups. Heart rate was significantly lower in ICI mice compared to non-ICI controls (CON: 661 ± 7.79 bpm vs. ICI: 624 ± 6.84 bpm; −5.59%; *p* < 0.05; Fig. 2e). Fractional shortening at euthanasia was significantly worse in the ICI mice compared to non-ICI controls (CON: 60.97 ± 3.91 vs. ICI: 47.35 ± 5.16; −22%; *p* < 0.05; Fig. 2f). Additionally, negative change in fractional shortening from baseline to endpoint was significantly larger in the ICI group (−9% vs. −29%; *p* < 0.05; Fig. 2g). Two-way repeated measures ANOVA also revealed a significant decline in fractional shortening from baseline (66.64 ± 4.33) to endpoint (47.35 ± 5.16) in the ICI group (*p* < 0.05) but not in the control group (Fig. 2h). Overall, ICI showed significant worsening in fractional shortening as a measure of cardiac function from baseline to endpoint and compared to controls, indicating that ICI administration led to declines in cardiac function.

### ICI treatment is associated with dysfunctional metabolism and muscle wasting

Cardiac tissue homogenates were analyzed for the protein levels associated with metabolism (AKT, FoxO1, FoxO3a; Fig. 3), and muscle wasting (Atrogin1, MuRF1; Fig. 4), to identify if metabolic and/or muscle wasting pathways are dysregulated following ICI treatment. Total protein expression of AKT, FoxO1 and FoxO3a was normalized to GAPDH and did not show significant differences between groups. The phosphorylated isoforms of AKT (i.e., P-AKT, active isoform) and FoxO1 and FoxO3a (i.e., P-FoxO1/3a, inactive isoform), were analyzed in cardiac tissue and normalized to total AKT, FoxO1 or FoxO3a. While not statistically significant, unpaired Student’s T-test revealed that P-AKT/AKT levels were lower in the ICI group compared to the CON group (Fig. 3b). Additionally, both P-FoxO1/FoxO1 and P-FoxO3a/FoxO3a levels were greater in the ICI compared to CON mice (Fig. 3c, d), with a statistically significantly greater levels of P-FoxO1/FoxO1 in the ICI group compared to the CON group (*p* < 0.05; Fig. 3c). These data suggest that pathways associated with metabolism may be dysregulated in ICI treated mice.

Further, cardiac tissue homogenates were analyzed for the expression of Atrogin1 and MuRF1. Both proteins are associated with muscle wasting cellular signaling pathways. Unpaired Student’s T-tests revealed statistically significantly greater protein expression of both MuRF1 (*p* < 0.05; Fig. 4b) and Atrogin1 (*p* < 0.05; Fig. 4c) in the ICI group compared to the non-ICI control group, indicative of dysregulated muscle wasting signaling pathways following ICI treatment. Combined with the results of the metabolic protein expression, these data suggest that pathways of metabolism and muscle wasting are dysregulated following ICI treatment, indicating dysfunctional protein homeostasis characterized by upregulated protein degradation (MuRF1, Atrogin1) and downregulated protein synthesis (P-AKT, P-FoxO1/3).

### ICI treatment is associated with abnormally enhanced autophagy

To further investigate how ICI treatment may negatively affect signaling pathways and lead to cardiotoxicity, the expression of autophagic flux was analyzed (Fig. 5), specifically focusing on early- and late-phase autophagy. Fluorescent microscopy was used to analyze autophagic flux in LC3 transgenic mice of each group, identifying different conformational stages of the dual LC3-GFP-RFP reporters in cardiac tissue. By fluorescent microscopy, early phase autophagosomes appear as yellow puncta (yellow puncta = RFP + GFP) while late phase autophagolysosomes appear as red puncta (red puncta = RFP; GFP signal is quenched by lysosomes, leaving behind only the RFP signal). Unpaired Student’s T-test revealed that there was a statistically significant difference in both early phase (yellow) puncta (autophagosomes) and late phase (red) puncta (autophagolysosomes) expression (Fig. 5a), with ICI mice expressing significantly more yellow (*p* < 0.05; Fig. 5b) and red puncta (*p* < 0.05; Fig. 5c) compared to non-ICI control mice. While not statistically significant, additional Western Blot analysis of autophagy-associated p62 protein levels showed a trend of decreased p62 levels in ICI treated hearts, potentially suggesting increased p62 degradation and enhanced autophagy. These data suggest that both early and late stages of autophagy were enhanced following ICI treatment, suggesting upregulated autophagic degradation pathways.

## Discussion

Immune checkpoint inhibitors (ICIs), first approved by the FDA in 2011 for the treatment of metastatic melanoma, are a relatively new anti-cancer immunotherapy that can initiate a biological immune response to fight cancer cells^28^. Despite being relatively new, ICIs have led to significant improvements in long-term remission rates and are associated with fewer side effects compared to other therapies (i.e., chemotherapy). ICIs aid in overcoming tumor-induced immunosuppression (weakening of the immune system) by blocking immune checkpoints that are frequently manipulated by tumor cells^29^. This ICI-induced blockade initiates biological immune responses to fight and ultimately destroy cancer cells^7^. However, despite the positive clinical outcomes on cancer progression, a rising number of ICI-treated cancer patients develop ICI-induced cardiotoxicity^8–10^. Cardiotoxicity is a rare, but potentially fatal side effect of ICIs^30^. While clinical cases of ICI-associated cardiotoxicity are rising rapidly, mostly affecting ICI-treated lung cancer patients^11^, clinical manifestations and underlying molecular mechanisms of this phenomenon are largely unknown. Due to the central function of ICIs blocking immune checkpoints and leading to infiltration of cytotoxic CD8 + T cells into the target tissue (i.e., tumor)^30^, it is highly speculated that immune cell infiltration and increased release of pro-inflammatory cytokines may play a critical role in the development of ICI-associated cardiotoxicity. Johnson et al. identified common infiltrating T cell populations in cardiac and tumor tissue postmortem of two ICI-treated cancer patients with fatal myocarditis^31^. While immune-mediated mechanisms are likely central to ICI cardiotoxicity, the underlying pathological immune-mediated and other involved mechanisms remain unclear. Therefore, the interest of this current study was to explore other pathways that may also be involved in ICI cardiotoxicity but not primarily associated with immune function. To further characterize ICI-induced cardiotoxicity in this study, female mice were treated with ICI treatment (i.e., anti-PD-1). The administered protocol has been used by previous labs and is accepted as a clinically relevant ICI treatment model^13,24,25^. In agreement with these studies, tumor data from this study showed that ICI treatment led to significant reductions in Lewis Lung Carcinoma tumor growth based on estimated tumor volume and relative tumor mass and therefore supports the efficacy and clinical relevance of the chosen ICI administration protocol.

While ICI-induced cardiotoxicities remain mostly undetected and underdiagnosed, if detected, electrocardiography is the primary diagnostic measure in the clinical setting^8,12^. However, while electrocardiography is a useful tool to identify abnormalities in electrical signal, cardiac structural changes remain difficult to detect using this diagnostic assessment. Pilot and exploratory studies utilizing echocardiography as an early diagnostic tool in ICI-treated cancer patients have noted an ease of application for assessing cardiac structure and function and high accuracy of detecting subclinical cardiac changes *via* echocardiography. However, research and clinical data available on echocardiographic diagnostic assessments of ICI-treated patients are limited^32,33^. Novel preclinical findings of this study suggest to consider expanding the clinical application of echocardiography as a diagnostic and clinical surveillance measure for early detection and identification of cardiac structural and functional changes in ICI-treated patients. In this study, mice treated with ICI experienced significant thinning of posterior walls and dilation of the left ventricle during systole and diastole, as well as significantly reduced fractional shortening, suggesting that the ICI protocol was capable of eliciting cardiac remodeling and dysfunction consistent with a phenotype of myocarditis and dilated cardiomyopathy^34^. Current cardio-oncology clinical practice guidelines by the European Society of Cardiology recommend transthoracic echocardiograms at baseline only in high-risk ICI-treated patients (e.g., ICI combination therapy)^35^. However, *all* ICI-treated mice in this study experienced these abnormalities in cardiac structure and function to some degree, demonstrating that (1) actual clinical incidence rates of ICI-cardiotoxicity are likely severely underreported due to the limited utilization of echocardiographic diagnostic assessments and (2) ICI monotherapy alone may be able to induce cardiotoxicity. Therefore, findings of this study agree with others suggesting that all patients treated with any ICI treatment strategy – not just those deemed high risk – should be considered to receive echocardiography assessments as a clinical surveillance assessment starting at baseline. Given the ease of application and widespread availability of echocardiography equipment, employing baseline and surveillance echocardiography would allow for early detection of cardiac abnormalities (i.e., changes in ejection fraction, left ventricular dilation) due to ICI treatment^32^.

While the underlying cardiac metabolic and muscle wasting signaling pathways affected by ICI treatment have not been evaluated yet, similar phenotypes of dilated cardiomyopathy have been observed in preclinical and clinical studies of cancer-mediated cardiac cachexia (wasting of the cardiac muscle due to cancer) and chemotherapy-induced cardiotoxicity. These wasting phenotypes have been associated with abnormal metabolism, characterized by an imbalance of protein synthesis and degradation^36–38^. The main pathways affected during chemotherapy-induced cardiotoxicity are the AKT/FoxO pathway, critical for protein synthesis, cardiac protection and cell survival^39^, and the ubiquitin-proteasome and autophagy-lysosome degradation systems (*via* expression of MuRF1 and Atrogin1)^15,40,41^. Therefore, expression of proteins associated with these anabolic and catabolic pathways was analyzed in this study to explore the involvement in ICI-induced cardiotoxicity. While not statistically significant, mice treated with ICI in this study showed decreased phosphorylation of AKT and therefore inhibition of AKT and downregulation of the anabolic AKT pathway. Additionally, phosphorylation of FoxO1 was significantly upregulated compared to non-ICI controls. The molecular mechanisms of FoxO1 in the heart are very complex as it is heavily involved in many aspects of protein homeostasis^42^. Under normal physiological conditions, AKT can phosphorylate FoxO1 and thereby inhibit the translocation to the nucleus and inactivate FoxO1^43^. When dephosphorylated, FoxO1 can translocate to the nucleus where it binds to transcription factors. Target genes of FoxO1 include Atrogin1 and MuRF1. Thus, activated FoxO1 can indirectly increase the activity of degradation pathways, such as the autophagy-lysosome and ubiquitin-proteasome systems, by binding to transcription factors in the nucleus and initiating the expression of target genes^43,44^. More recently, it was shown that acetylation and deacetylation of FoxO1 can change the activity status of FoxO1 in the cytosol, suggesting that even phosphorylated (“inactive”) FoxO1 can be activated outside of the nucleus and bind directly to proteins associated with degradation processes^45–47^. This alternative mechanism could be of specific interest for future investigations when looking at the findings of this exploratory study. Additionally, previous studies have found that pathophysiological phosphorylation of FoxO1 in cardiomyocytes following doxorubicin chemotherapy resulted in apoptosis and chemotherapy-induced cardiotoxicity^48^. Therefore, in line with previous studies investigating chemotherapy-induced cardiotoxicity and potential alternate mechanisms that can activate phosphorylated FoxO1, ICI treatment in this study seems to induce cellular changes in metabolic pathways *via* the AKT/FoxO1 pathway, resulting in ICI-induced cardiotoxicity.

Aligning with these data on ICI-induced changes in AKT and FoxO1 protein expression, expression of proteins associated with cellular degradation pathways (i.e., MuRF1 and Atrogin1) were also affected by ICI treatment in this study. Both MuRF1 and Atrogin1 cardiac protein expressions were significantly upregulated (*p* < 0.05) in the ICI group compared to non-ICI controls. Previous research has repeatedly shown that these molecules, specifically MuRF1, are involved in chemotherapy-induced cardiotoxicity (i.e., chemotherapy-induced cardiac atrophy). Findings of this study suggest that ICI treatment upregulated the cardiac expression of MuRF1 and Atrogin1 similar to what has been described in chemotherapy-induced cardiotoxicity. Interestingly, pharmacological inhibition of MuRF1 has been shown to reduce or even reverse cardiac atrophy and preserve cardiac function in these cardiotoxicity models^49,50^, suggesting that MuRF1 may also serve as a therapeutic target in ICI-induced cardiotoxicity. To further analyze this interplay and involvement of degradation pathways, the autophagy-lysosome pathway as an important degradation pathway likely involved in chemotherapy- and ICI-induced cardiotoxicity was also analyzed in this study. While cardiomyocyte autophagy remains an understudied mechanism, preliminary preclinical evidence suggests a context-dependent role of autophagy^51^. Specifically in chemotherapy-induced cardiotoxicity, therapeutic upregulation of autophagy reverses or suppresses chemotherapy-induced cardiotoxicity and promotes cardiomyocyte survival in rodents^52–54^. However, other in vitro and in vivo studies have found conflicting evidence that exposure to chemotherapy itself induced an upregulation of autophagy and resulted in cell injury and death in doxorubicin-treated isolated cardiomyocytes and hearts of doxorubicin-treated animals^40,55,56^. Therefore, while autophagy seems to play a role in the pathophysiology of chemotherapy-induced cardiotoxicity, a lack of consensus is evident regarding the specific role and alterations of autophagy in chemotherapy-induced cardiotoxicity.

To our knowledge, the role of autophagy in ICI-induced cardiotoxicity has not been evaluated yet. Autophagic flux analyses of CON and ICI mice in this study revealed that ICI treated mice showed significantly upregulated early- and late-phase autophagy in cardiac tissue due to ICI treatment. These findings are in line with the increased expression of MuRF1 and Atrogin1 in ICI mice and suggest that the initiation phase and the final degradation phase of the autophagy-lysosome system are upregulated in mice hearts following a 4-week ICI treatment protocol, likely one mechanism involved in the cardiac remodeling and dysfunction observed in ICI-treated mice.

Overall, results of the exploratory analyses of underlying pathological signaling pathways that could be dysregulated by exposure to ICI treatment revealed that our ICI model induced dysregulations in metabolism (P-AKT/AKT, P-FoxO1/FoxO1) and muscle wasting (MuRF1, Atrogin1), characterized by downregulation of protein synthesis pathways and upregulated protein degradation pathways (autophagic flux). The underlying cellular imbalance of catabolic and anabolic pathways likely explains the cardiac structural and functional changes observed *via* echocardiography in ICI mice compared to non-ICI controls. While this exploratory study on a small sample size of ICI-treated mice sheds light on potential underlying metabolic and muscle wasting pathways associated with ICI-induced cardiotoxicity, future research should investigate the interplay between the identified metabolic and muscle wasting pathways with immune-mediated mechanisms in a larger sample size and their synergistic involvement in ICI-cardiotoxicity. This study provides the first step to determine the isolated effects of ICI treatment on cardiac remodeling and dysfunction in healthy female mice. Future investigations should aim to identify whether immune-metabolic remodeling and ICI-associated cardiotoxicity might differ in the presence of tumor burden and in male counterparts (i.e., influence of hormonal status on metabolic and/or muscle wasting pathways).

## Conclusion

In conclusion, this study sought to further identify ICI-associated cardiac structural and functional changes and determine pathological cellular signaling pathways in cardiac tissue that are dysregulated following ICI treatment. In this model, ICI-induced cardiotoxicity was characterized by cardiac remodeling and dysfunction with a phenotype similar to dilated cardiomyopathy. Additionally, ICI treatment was associated with a disruption of protein homeostasis characterized by downregulation of protein synthesis and upregulation of muscle wasting and autophagy. Therefore, this study adds novel and impactful insight to the still unclear characteristics of ICI-induced cardiotoxicity. While many question remain regarding the translatability of these findings and the cellular mechanisms involved in cancer patients suffering from ICI-induced cardiotoxicity, our study supports the critical need to further investigate underlying pathological mechanisms associated with side effects of immunotherapies to optimize clinical treatment of cancers.

## Supplementary Information

Below is the link to the electronic supplementary material.

Supplementary Material 1

## Data Availability

The datasets used and/or analyzed during the current study are available from the corresponding author on reasonable request.

## Abbreviations

CON: Control group
ICI: Immune checkpoint inhibitor ; immune checkpoint inhibitor treated group
irAE: Immune–related adverse event
LLC: Lewis lung carcinoma
LVD: Left ventricular diameter
NSCLC: Non–small cell lung cancer
PD: 1–programmed cell death 1
PW: Posterior wall
SW: Septal wall
