# Supplementary material for: Anti-PD-1 immune checkpoint inhibitor-induced cardiotoxicity is associated with dysfunctional metabolism, muscle wasting and autophagy
Source: Sci Rep. 2026 Jan 15;16:4240. doi: 10.1038/s41598-025-34379-4 (PMC12858808; doi:10.1038/s41598-025-34379-4)
Supplement: Supplementary file 1 — Supplementary Material 1 [file 41598_2025_34379_MOESM1_ESM.pdf]

Nature Scientific Reports Manuscript

Title: *Anti-PD-1 Immune Checkpoint Inhibitor-Induced Cardiotoxicity is Associated with Dysfunctional Metabolism, Muscle Wasting and Autophagy*

Supplementary Figure. 1. In Vivo Echocardiography at Baseline of Study. Left ventricular diameter (a, d) and wall thicknesses (b, c, e, f) at end-diastole (a-c) and end-systole (d-f) represent an average of three consecutive cardiac cycles per mouse. CON, control group (n = 5); ICI, ICI treated group (n = 5); N = 10. Values are reported as mean ± SD.

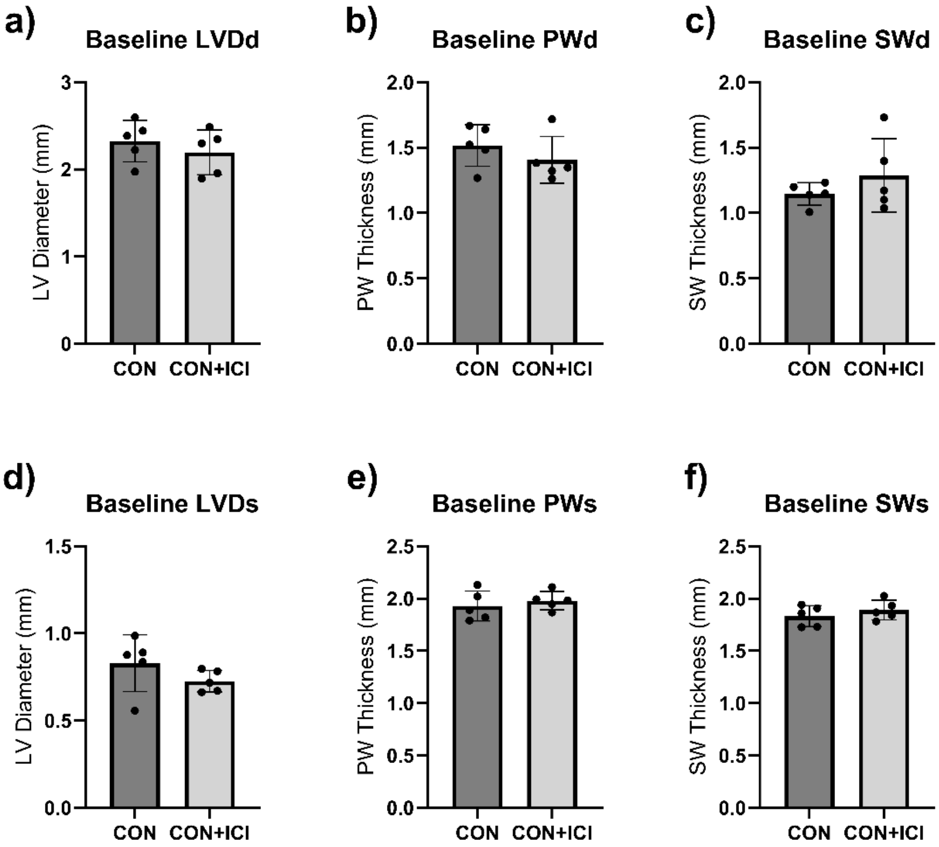

Supplementary Figure 2. Original western blots and western blot standard. A) Magic mark XP western protein standard was used for all western blots. B) Western blots were conducted on cardiac tissue for protein levels: P-AKT, AKT, P-FoxO1, FoxO1, P-FoxO3a, FoxO3a, MuRF1, Atrogin1, and GAPDH. Black horizontal lines indicate mice included in this study; red boxes indicate cropped representative images appearing in figures.

A) Magic Mark Protein Ladder

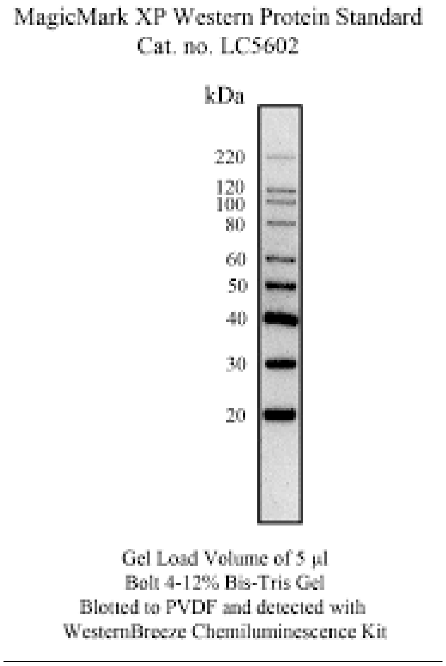

B) Whole Western Blot Images

P-AKT (60 kDa)

Exposure: 10sec

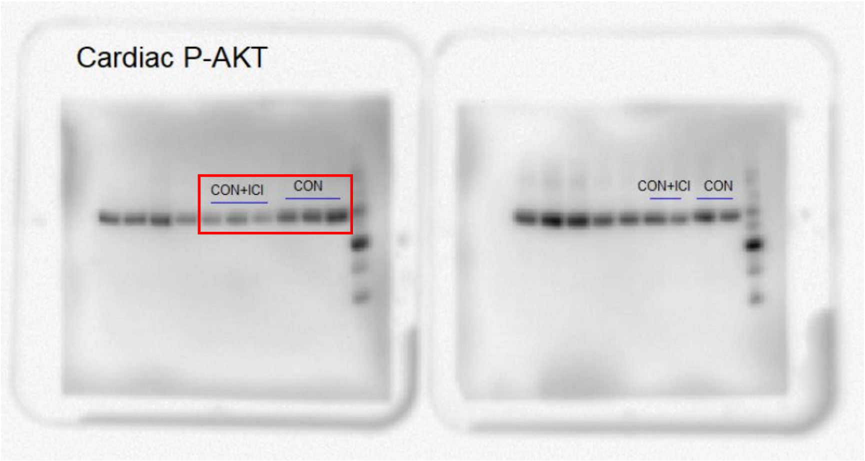

AKT (60 kDa)

Exposure: 30sec

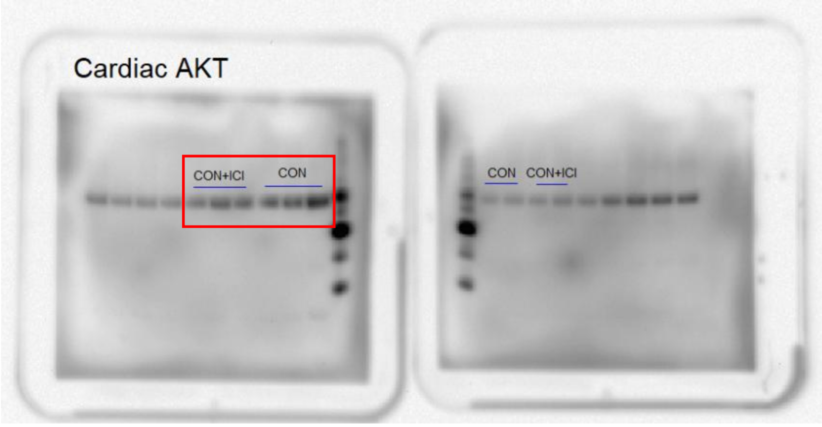

P-FoxO1/3a (78 to 82, 95 kDa)

Exposure: 240sec

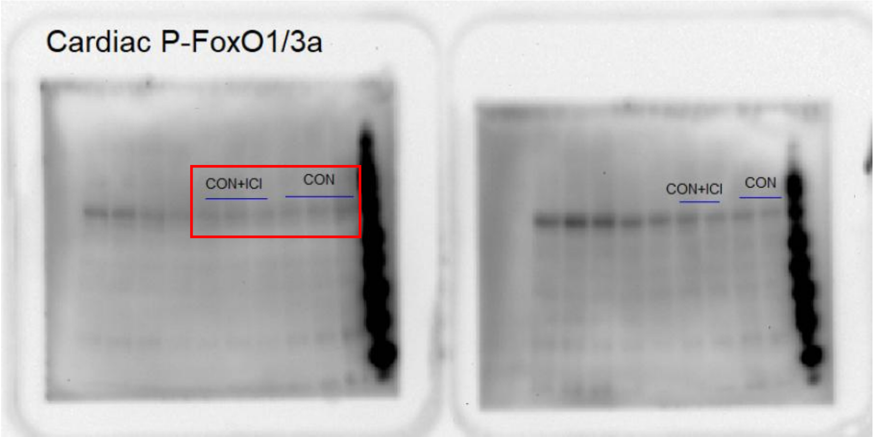

FoxO1 (78 to 82 kDa)

Exposure: 45sec

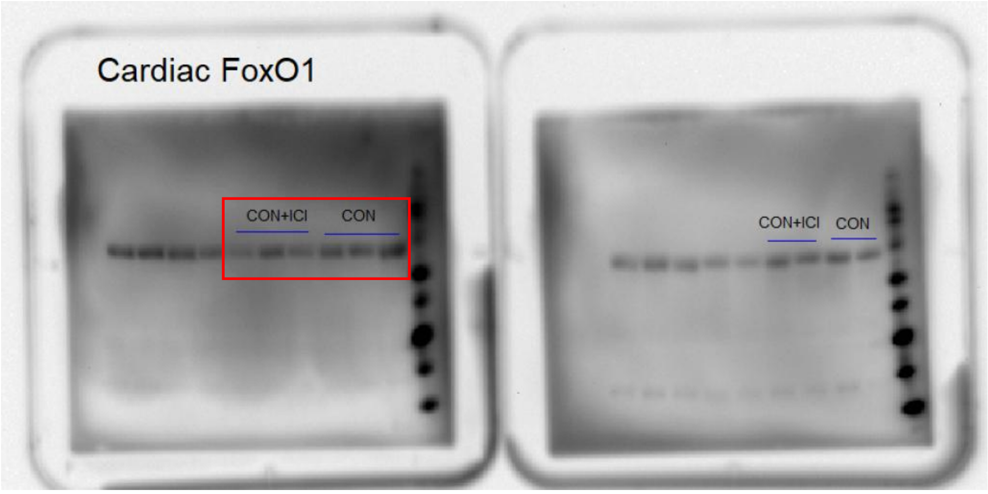

FoxO3a (82 to 97 kDa)

Exposure: 360sec

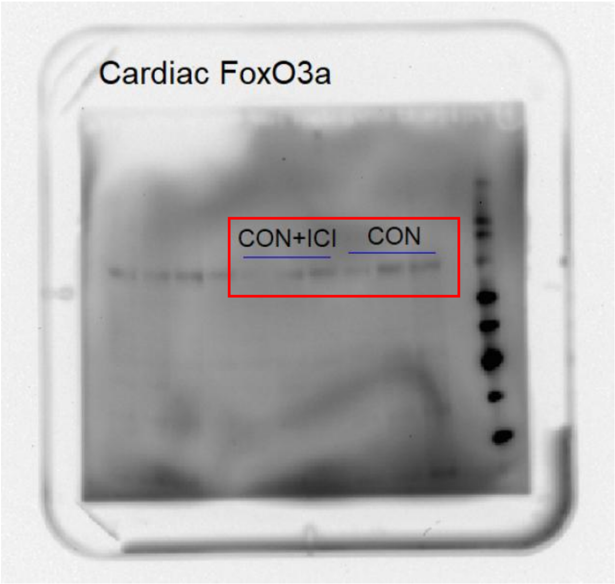

MuRF1 (40kDa)

Exposure: 8sec

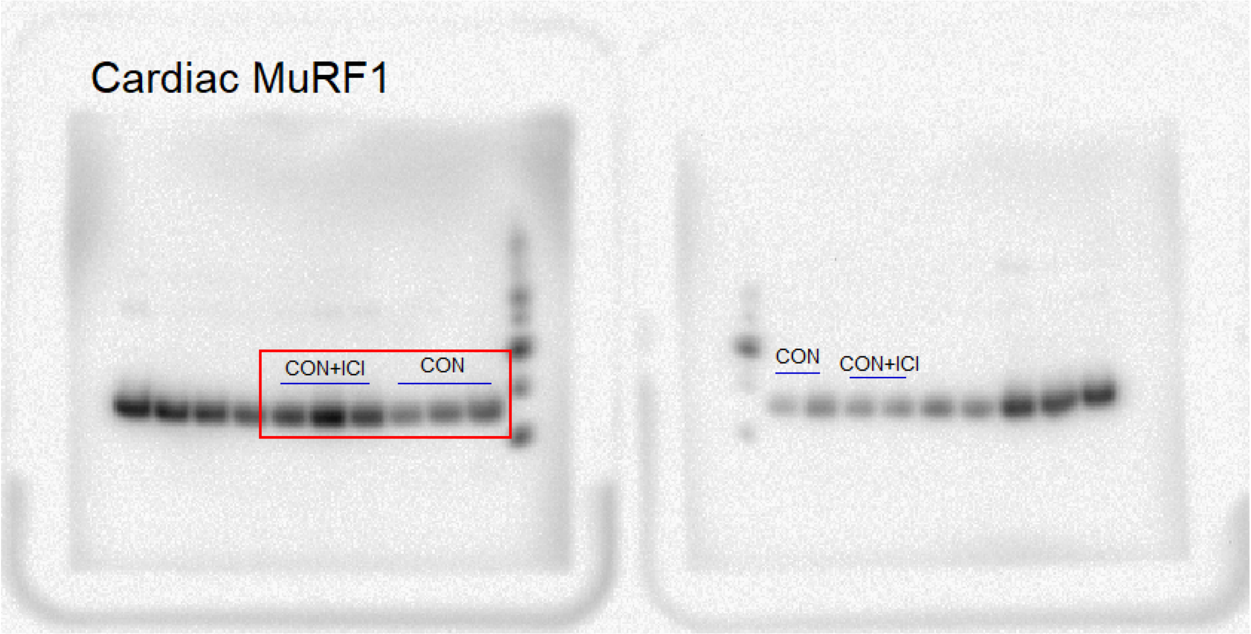

Atrogin1 (42kDa)

Exposure: 8sec

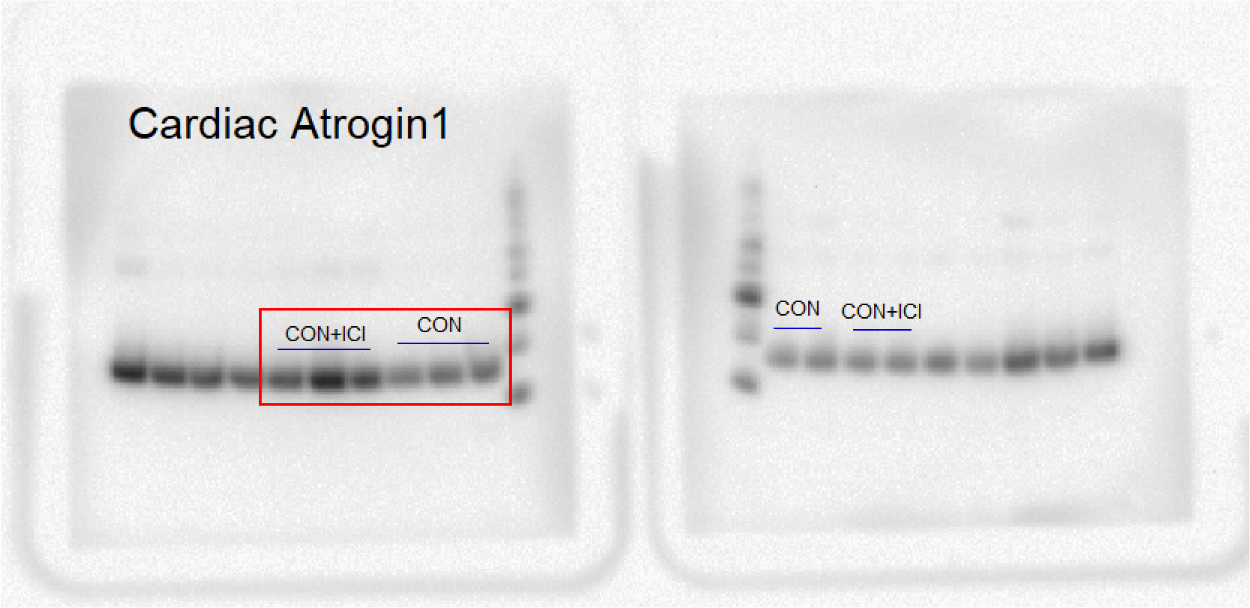

GAPDH (37kDa)

Exposure: 8sec

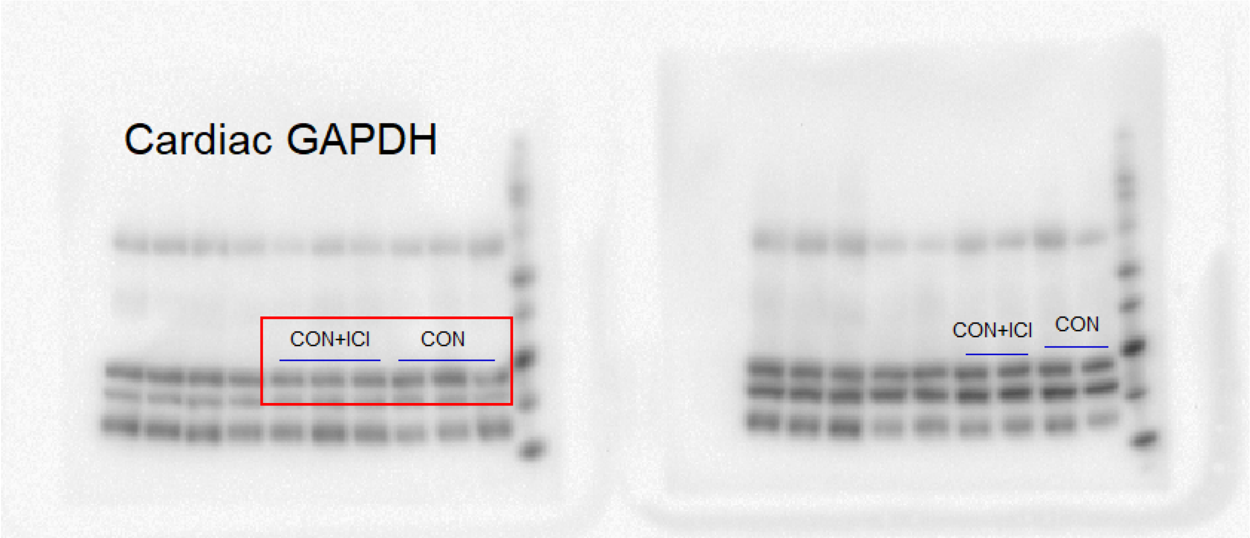
Cardiac GAPDH
CON+ICI
CON
CON+ICI
CON
